# Gas Transport in Glassy Polymers

**DOI:** 10.3390/membranes10120400

**Published:** 2020-12-07

**Authors:** Maria Grazia De Angelis, Giulio C. Sarti

**Affiliations:** Department of Civil, Chemical, Environmental and Materials Engineering, University of Bologna, I-40131 Bologna, Italy; giulio.sarti@unibo.it

This Special Issue of Membranes provides an updated and comprehensive overview of the state of fundamental knowledge on the fluid sorption and transport in glassy polymers, combining original experimental and modeling works, as well as reviews, prepared by renowned experts. Sophisticated experimental insights are given at the transport in glassy polymeric materials in challenging operative ranges such as in mixed gas conditions, in the presence of humidity and of strongly interacting species, as well as at high pressure and temperatures. A thorough investigation of the transport in complex novel structures, based on high performance polymers incorporating fillers of different types (Mixed Matrix Membranes, MMMs) is also presented. Macroscopic models suitable for the interpretation of competition effects during the mixed gas sorption in glassy polymers are analyzed, while a comprehensive review of the modeling of facilitated transport in membranes is presented. Finally, a complete review is provided of the most recent and effective molecular and multiscale models for the simulation of complex glassy polymeric structures and for the fluid sorption and transport therein. The present issue is mainly devoted to the applications of membranes for fluid separations, although the results obtained are valid in general.

The experimental analysis of transport in glassy polymers often requires dedicated techniques because experiments are time-consuming, due to the long relaxation times typical of glassy polymers, and they might show history-dependent properties that require appropriate pretreatment protocols. In many cases, connection with theoretical models is required to support the experimentation and the analysis of the results.

The experimental analysis is complicated when strong interacting species such as polar compounds are considered, for which specific models are required. In the paper by Mensitieri et al. [1], the sorption thermodynamics of CO_2_, H_2_O and CH_3_OH interacting with a glassy Poly (ether imide) (PEI) were studied; gravimetric analysis and vibrational spectroscopy were used for the collection of the data, and non-equilibrium thermodynamics modeling was applied for their interpretation. The molecular information gathered from the Fourier-transform infrared (FTIR) spectroscopic analysis is used to tailor thermodynamics modeling. The investigated penetrants display different interactions with the polymer, which reflects in different sorption thermodynamic properties. For the specific case of water, the outcomes from molecular dynamics simulations are compared with the results of the analysis.

The sorption of CO_2_ in PEI could be described with the NELF model in view of the weak interactions established with groups present on the polymer backbone. For the case of H_2_O sorption, data were interpreted using the Non Equilibrium Thermodynamics for Glassy Polymers-Non Random Hydrogen Bonding (NET-GP-NRHB) model in order to cope with specific H-bonding interactions occurring in the PEI–H_2_O system. FTIR spectroscopy revealed the occurrence of two water species, one self-interacting via H-bonding and the other cross-interacting with carbonyl groups of PEI, making it possible to adjust the H-bonding terms in the NET-GP-NRHB model. 

In glassy polymeric membranes for gas separation, membranes’ multicomponent effects have been proven to be particularly important, especially because of competition between penetrants, which affects sorption and sorption-selectivity. Mixed gas effects can completely reverse the performance of membranes characterized under pure gas conditions only.

In the paper by Genduso et al. [2], the nonideal behavior of CO_2_-CH_4_ mixtures was elucidated in 6FDA-mPDA, a polyimide of remarkable performance. To quantify the deviation from ideality, sorption and diffusion contributions to permeation were decoupled. Experimental data of mixed-gas solubility revealed a decrease in both CO_2_ and, more markedly, CH_4_ solubility due to mixture effects. CO_2_ versus CH_4_ mixed-gas solubility coefficients of 6FDA-mPDA and other glassy polymers previously studied follow a linear trend regardless of equilibrium concentration. Since the CO_2_/CH_4_ solubility selectivity of 6FDA-mPDA improved under mixed-gas conditions, the observed decline in mixed-gas permeability selectivity from the corresponding ideal values cannot be attributed to competitive sorption, as frequently assumed in the literature, but rather to a depression of the size-sieving capability of 6FDA-mPDA induced by the presence of CO_2_ in the polymeric film matrix. The authors performed kinetic measurements which reinforced the idea that CO_2_ addition lowers the effective diffusion coefficient of CH_4_ in the polymer.

In the scientific literature on gas separation membranes, most data are reported in pure gas conditions, at room temperature and relatively low pressure. However, many separations would preferably occur at high temperatures and pressures, such as in the case of pre-combustion CO_2_ capture where H_2_/CO_2_ separation is relevant, and natural gas treatment where the CO_2_/CH_4_ mixture is involved. In addition, temperature and pressure have a dramatic impact on the membranes’ performance, because the selectivity can decrease or even reverse with temperature, and the high pressure of mixtures containing CO_2_ causes plasticization.

In the paper by Lasseguette et al. [3], the transport properties of CO_2_, N_2_, CH_4_ and H_2_ in a Polymer of Intrinsic Microporosity (PIM-EA(H_2_)-TB), with high CO_2_ and H_2_ permeability and good ideal selectivity over N_2_, were determined for upstream pressures up to 20 bar and temperatures up to 200 °C. No increase in CO_2_ permeability due to plasticization was noted over the range of pressure tested. The permeability coefficient of N_2_, CH_4_ and H_2_ increase with temperature, while for CO_2_ the permeability decreases with temperature, due to its high solubility, which is an exothermic process. Therefore, the separation performance of PIM-EA(H_2_)-TB for H_2_/CO_2_ is reversed at high temperature and maintained also at high pressure. This suggests that, after further development to enhance absolute selectivity of H_2_ over CO_2_, PIMs could become good candidates for membrane materials for use in pre-combustion CO_2_ capture.

A significant interest was created in the last 20 years around Mixed Matrix Membranes (MMM), namely polymers to which particles of different structure and morphology were added, with the aim of improving their performance in fluid separations. The glassy polymers, due to their rigidity, are particularly suitable for incorporation of such fillers, which normally reduce the flexibility of softer materials like the rubbery ones. The interactions between the polymer and the filler play an important role in determining the final performance.

In the paper by Dai et al. [4], Poly(1-trimethylsilyl-1-propyne) (PTMSP), a polymer with an exceptionally high gas permeation rate but a serious aging problem and low selectivity, was combined with different selective fillers with the aim of increasing its selectivity. The gas permeation of the hybrid membranes was evaluated using a mixed gas permeation test with the presence of water vapour to simulate the flue gas conditions. The addition of ZIF-L improves the CO_2_/N_2_ selectivity at the expenses of CO_2_ permeability, while the addition of TiO_2_, ZIF-7 and ZIF-8 increases the CO_2_ permeability but the CO_2_/N_2_ selectivity decreases. The hybrid membranes are characterized by high thermal stability, while the poor affinity with the polymer phase causes the formation of interfacial voids. The solvent used for the membrane preparation has a strong effect on the final performance, as is usually the case with glassy polymers. For all the hybrid membranes, increasing the water vapour content in the gaseous streams lowers the CO_2_ permeability.

Escorihuela and coworkers [5] studied the effect of adding different fillers on various polyimides suitable for gas and liquid separations, particularly for biogas upgrading and organic solvent nanofiltration (OSN). The most promising result was obtained for Matrimid^®^—10 wt.% BaCe_0.2_Zr_0.7_Y_0.1_O_3_ (BCZY) MMM, which showed improvement in CO_2_ and H_2_O permeabilities accompanied by increased CO_2_/CH_4_ selectivity. It was also observed that, in general, the inorganic fillers could produce small rigidification in the polymer matrix, although they do not exhibit higher T_g_. Regarding the temperature effects, some changes were observed in the activation energy of the process, while the incorporation of inorganic fillers did not significantly affect the permeation mechanism determined by the polymer transport properties. Water permeability was first reported for several polyimides and MMMs of inorganic particles with polyimides, reaching relatively high values.

In the review by Golemme and Santaniello [6], the focus is on MMMs formed by molecular sieves dispersed in perfluorinated glassy polymers. First, the issue of compatibilization of ceramic molecular sieves with the polymers is considered, examining the effect of the surface treatment on the gas transport properties of the filler. Then, the preparation of the defect-free hybrid membranes and their gas separation capabilities are described. Finally, modelling of the gas transport properties of the perfluoropolymer MMMs is reviewed, with a four-phase approach implemented in the frame of the Maxwell model and finite element simulation. The four-phase approach is a convenient representation of the transport in MMMs when more than one single interfacial effect is present. Grafting perfluorinated tails on the outer surface of zeolitic molecular sieves improved the surface permeability and allowed the preparation of defect-free MMMs with perfluoropolymers.

Macroscopic models for describing fluid transport in glassy polymers are computationally inexpensive but their accuracy can be limited by the absence of the experimental information required to parametrize them. Two important cases of study are reported: the simulation of mixed gas sorption, and the facilitated transport process.

As pointed out above, the mixed gas sorption in glassy polymers is complicated by the presence of competition effects and it is important to rely on predictive models which could estimate the mixed gas performance in the absence of specific experimental data. The paper by Ricci and De Angelis [7] aims at assessing the performance of a model used to predict the mixed-gas solubility and selectivity of glassy membranes, the Dual Mode Sorption (DMS) model in its multicomponent extension.

The data of CO_2_/CH_4_ mixtures in three high free volume glassy polymers, PTMSP, PIM-1 and tetrazole-modified PIM-1 (TZ-PIM), were taken as test cases. These systems exhibit deviations from the ideal pure-gas behavior, especially due to competition. The DSM model parameters retrieved from the best fit of pure-gas sorption isotherms provided a good qualitative picture of sorption in mixed-gas conditions, displaying a reduction in solubility that was experimentally observed and due to competition. A sensitivity analysis carried out on the DMS model parameters revealed that a small uncertainty in the pure-gas data propagates greatly in the mixed-gas calculation, limiting the quantitative accuracy of the mixed-gas prediction in the absence of experimental mixed-gas data to use for validation. The authors concluded that the DSM model is a useful tool for a first estimate of the mixed-gas effects, due to its simplicity, but that for quantitative accuracy one might resort to other models such as the Non Equilibrium Lattice Fluid (NELF) model.

The Facilitated Transport (FT) process is one of the most promising processes when it comes to CO_2_ capture with membranes, because the reaction taking place between diffusing CO_2_ and certain aminic species present in the membrane boosts the CO_2_ selectivity and permeability. The process is composed of pure diffusion and chemical reaction between solute and carrier and requires a specific modeling approach. The review by Rea et al. [8] elucidates the various models devoted to explaining the FT mechanism in the two main families of carrier-mediated membranes: the mobile carrier (MC) systems where the carrier is free to diffuse across the membrane, and the fixed site carrier (FSC) where the carrier is fixed to the polymeric backbone.

For the MC systems, the models by Teramoto and by Morales-Cabrera et al. seem to be the more flexible ones as they can be applied in a wide range of operative conditions, without strong assumptions but with the numerical solution of a system of equations. The simpler models, provided by Smith and Quinn, Noble et al. and Jeema and Noble, have a good descriptive ability coupled with a very simple mathematical form, but they are only valid under certain assumptions. For the FSC systems, interesting approaches are those by Noble (1992), Kang et al. (1996) and Zarca et al. (2017). Despite the number of modeling tools available, the main issue is the knowledge of the physico-chemical parameters of the models, which are difficult to determine experimentally.

The modeling of fluid transport in glassy polymers can be made less reliant on experimentally-derived parameters by using more predictive modeling tools like atomistic simulations. However, glassy materials are challenging for such methods, due to the long relaxation times that make the calculations computationally expensive. The review by Vergadou and Theodorou [9] aims at giving an overview of the most recent efforts dedicated to the development of molecular and multiscale methods for the simulation of sorption and diffusion in glassy polymers. The basic concepts of equilibrium and non-equilibrium Molecular Dynamics (MD) and Monte Carlo (MC) techniques for simulating polymers and sorption and diffusion of small molecules are introduced. For diffusion, the focus is placed on multiscale methods that adopt infrequent event analysis of elementary diffusive jumps, such as the Transition State Theory (TST) and the Transition Path Sampling (TPS) Method. Indeed, penetrant jumps between sorption sites in glassy polymers cannot be simulated with MD, due to the excessively long computational times required. Multiscale methods such as those based on coarse-graining/reverse mapping for the generation of realistic polymeric configurations and for the sorption and diffusion phenomena in polymers per se are also discussed. Recent approaches designed to cut down on the computational cost, such as mesoscopic kinetic MC techniques that utilize atomistic information and coarse-grained MD simulations with appropriate time mapping, are discussed. Finally, open challenges and future perspectives of molecular simulations of transport in dense membranes are illustrated.

In conclusion, it can be said that glassy polymers, and their use in applications where fluid transport is important, still have open issues that need to be investigated. We hope that this Special Issue provides inspiration to new and established scientists in the field, and a source of information about the different aspects of the problem that often need to be considered altogether.
